# Sampling efficiency of a protocol to measure Odonata diversity in tropical streams

**DOI:** 10.1371/journal.pone.0248216

**Published:** 2021-03-09

**Authors:** Joana Darc Batista, Victor Rennan Santos Ferreira, Helena Soares Ramos Cabette, Lourivaldo Amancio de Castro, Paulo De Marco, Leandro Juen

**Affiliations:** 1 Entomology Laboratory of Nova Xavantina, Universidade do Estado de Mato Grosso, Nova Xavantina, Brazil; 2 Laboratory of Ecology and Conservation, Pós-graduação em Ecologia, Universidade Federal do Pará and Goeldi Museum, Belém, Brazil; 3 Laboratory of Theory, Metacommunity & Landscape Ecology, Universidade Federal de Goiás, Goiânia, Brazil; Universidade Regional Integrada do Alto Uruguai e das Missoes, BRAZIL

## Abstract

Odonata can be sampled following different types of protocols. In Brazil, the most used protocol is the scanning in fixed areas method, where a 100-meter transect is delimited in one of the stream margins, subdivided into 20 segments measuring 5 meters. Despite being universally used, the methodological efficiency or limitations of this protocol for Odonata has never been tested. In this scenario, our objective was to assess the efficiency of the sampling protocol to measure the richness and composition of Odonata in three fundamental aspects: the time of sampling and sampling effort over time and space. We show that the best sampling efficiency was achieved in collections performed at noon, in transects measuring 100 meters, requiring at least two samplings in the same location, supporting the procedures traditionally adopted by many studies with the group. While comparing species composition, we did not see any implication between the different treatments on the capture of the local species pool. However, we highlight and discuss some possible methodological flaws when using this protocol to sample specific Odonata groups. We believe the results obtained are fundamental in the inventory of species and to conduct future studies, as well as to aid conservative measures that use the order Odonata as a tool for environmental monitoring.

## Introduction

The number of scientific studies addressing the Odonata order has increased in the last years [1, 2]. To get the idea of this growth, from 1945 to 2021, we found a total of 4.713 articles when searching the term “Odonata” as a filter on the “Web of Science” platform. The contribution of these articles is more consistent in different research areas in ecology, especially modelling, macroecology, and intra/interspecific relationships [1]. The constant use of this group as model organisms in ecological studies is due to singular characteristics found in the order Odonata, such as: sensitivity to environmental change [3, 4]; specific ecophysiological requirements, where environmental factors directly influence their daily activities and even the community structure [5, 6]; amphibiotic life cycle, allowing to test hypotheses according to the instar [7, 8]; broad geographical distribution, where the diversity of the group are extremely high, whether in natural environments or anthropogenic environments [9–11].

Despite the notoriety and importance of the order Odonata in scientific studies, basic methodological questions remain unanswered or were partially assessed, which can generate wrong or methodologically unviable conclusions [12, 13]. For example, thermoregulation acts as a significant filter in the distribution and activity of Odonata [6], where there are species that are more active in warm environments and with abundant solar radiation (ectotherms), and species that can produce heat internally, depending less on the temperature of the environment (endotherms) [5]. Therefore, since throughout the day there are variations in environmental characteristics (e.g., temperature and humidity) that are important to the dynamics of the community [11], when is the best time of the day to sample odonates? Furthermore, considering the different detectability of species [14, 15], be it by naturally low abundance or singular physiological and behavioural characteristics, how many samplings would be necessary to meet these specificities and, therefore, to sample the local diversity in a representative manner?

Regarding spatial issues, one of the major sampling problems in tropical lotic environments is the high environmental heterogeneity, whether due to natural variability of the substrates, the catchment, width, depth, flow, riparian vegetation, or the variation breadth of physical-chemical conditions [16]. All those factors are important, as they provide specific environments for the species. Additionally, it is predicted that the colonization and permanence of dragonflies have direct implications in the species-area relationship [17] and environmental heterogeneity [3, 4, 18]. Therefore, the larger and more variable the environment, the more habitats, resources, and more available niches are expected, causing dragonfly abundance and richness to increase possibly. Sampling protocols must be adapted to these variations, otherwise, the existing biodiversity might be underestimated.

Adult Odonata can be sampled following different types of protocols [3, 12, 19–21]. The existing convergence among the methods is that they all have standardized efforts, be it by time, space, or both. In tropical forests a widely applied protocol is an adaptation of the method initially proposed by Pollard [22] and adapted for dragonflies by De Marco [19, 23] (see more in [12, 24]). According to the protocol, a 100-meter stretch must be set at the stream margin, and all individuals seen in an interval of 1 hour must be registered. To facilitate the sampling process, as well as to create sub-samples that are important for statistical analyses, it is recommended to divide the transect into 20 sub-samples measuring five meters each. This protocol was recently redesigned and discussed by Cezário et al. [25] proposed as a standard protocol for ecological studies of Odonata. It has been widely used in different environments [6, 12, 17, 26–31], although the methodological efficiency or limitation has never been tested.

Given this scenario, this study aimed was to assess the efficiency of the adult Odonata sampling protocol in three fundamental aspects:

- Sampling time, by evaluating the prediction that increased species richness will be found at the time of the day where the temperature at its peak, at 12:00 a.m. (noon). Additionally, we predict species composition will remain the same once the evaluated timespan does not include crepuscular species, only species with peak daytime activity.
- Sampling effort over the days. We believe there will be no significant difference in species richness and composition in relation to the number of days collected at the same site, provided the sampling is done at the time of highest activity for Odonata and that the climatic conditions are favorable for collection.
- Transect area size required for representative sampling. We expect an increase in Odonata richness as the sampled area increases, considering that larger transects increase the probability of contemplating different habitats and, therefore, collecting different species. However, we do not believe composition will change dramatically since dragonflies are organisms with a high dispersal ability, flying quickly along the stream.

## Materials and methods

### Study area

**Fig 1:**
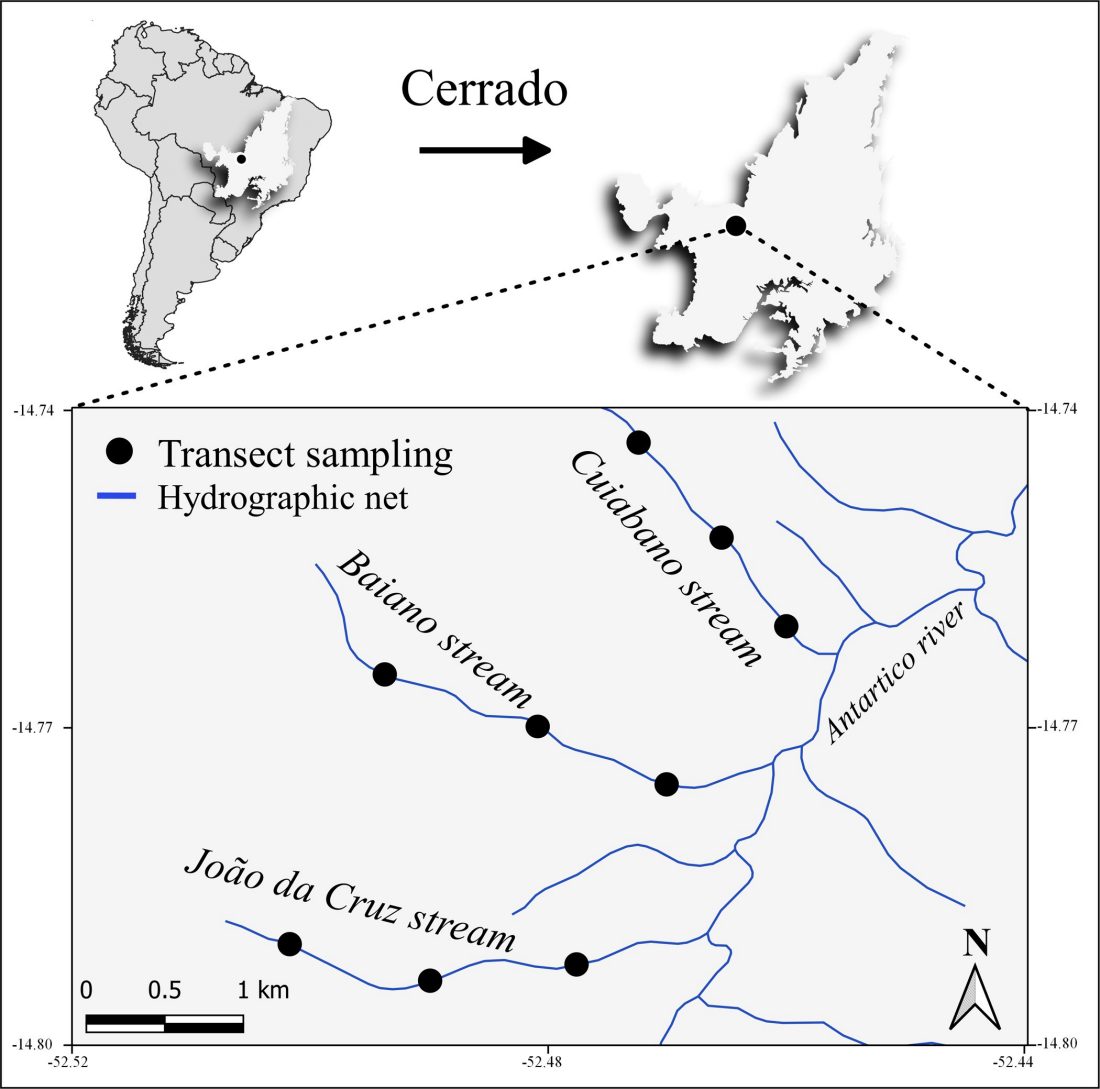
Study area. All streams sampled are in the Cerrado biome and belong to the Ribeirão Antártico Basin, municipality of Nova Xavantina, MT, Brazil.

The study was conducted in three streams of the Brazilian Savanna (Cerrado)–João da Cruz (CRJC), Baiano (CRB), and Cuiabano (CRCU)–located at the mean portion of the Ribeirão Antártico Basin, in the municipality of Nova Xavantina—MT (between the coordinates: 14°46’43”, 14°45’22” S and 52°29’07”, 52°27’29” W, with an average altitude of 300 meters) (Fig 1). We emphasize the low geographic distance between streams is important in the present study, as it allows for a greater similarity in the environmental conditions between the sampling sites, as well as connectivity between metacommunities, thus avoiding great differences in species diversity. According to the classification proposed by Strahler [32], the sampling sites are third-order streams, with an average width of approximately 1 to 2 meters in the initial and intermediate stretches, extending to three meters in the final stretches. All streams have an undisturbed and continuous gallery forest, as well as signs of a good conservation state. The vegetation beyond the riparian forest was previously covered by Cerrado vegetation. However, it was partially replaced by pasture, except for the Cuiabano stream, in which livestock activities were abandoned and, therefore, is in a regeneration phase. According to the Köppen classification, the region’s climate is Aw (tropical savanna), with two well-defined climatic seasons: a dry (from May to September) and rainy (October to April) season [33]. The average annual rainfall is 1.500 mm, and the average temperature is 27°C.

### Sampling and collection methods

**Fig 2:**
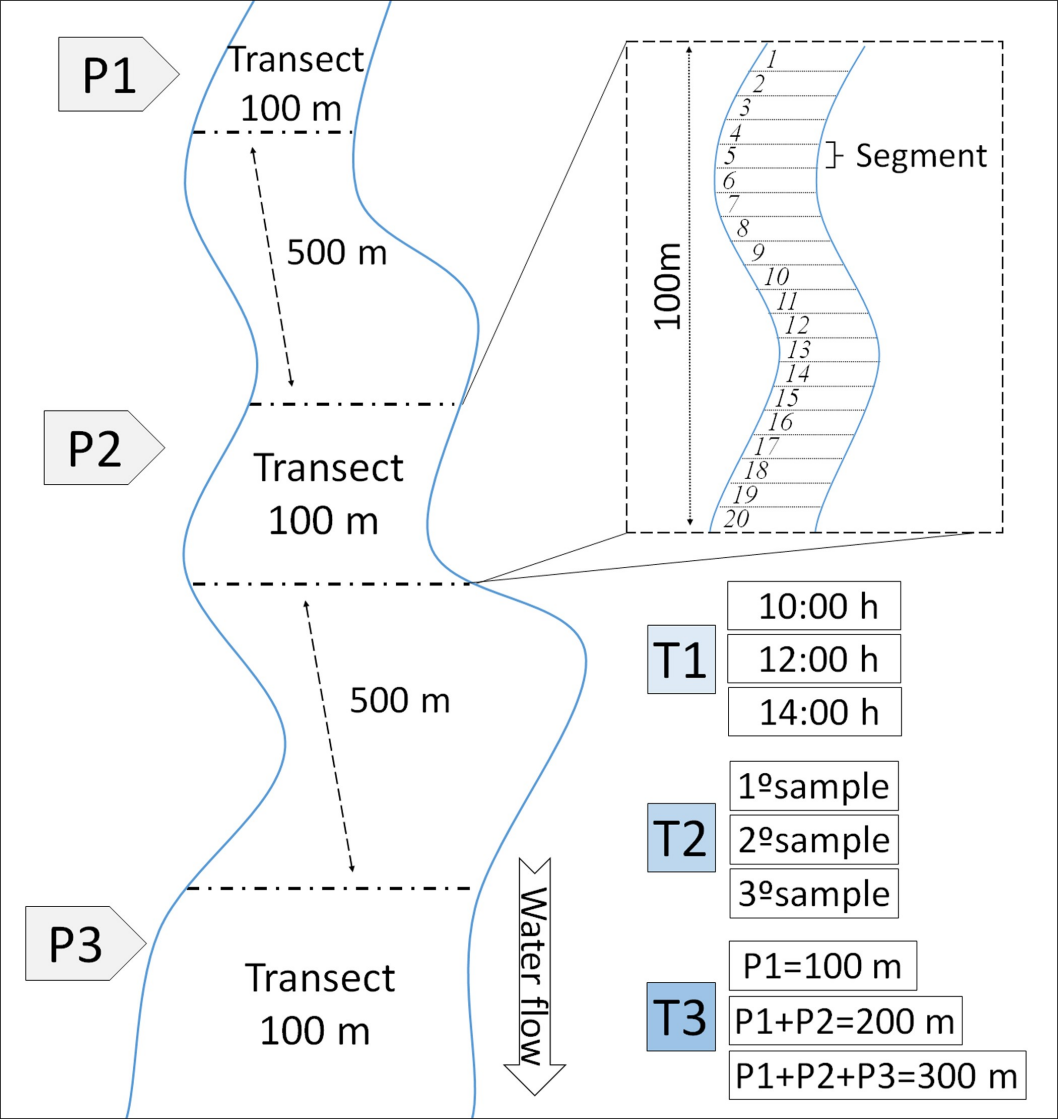
Sampling design. We evaluate the efficiency of the sampling protocol adapted to Odonata collection.

Sampling was carried out in June 2008, during the dry season, on sunny days, always with a temperature above 25°C. As mentioned, the method of collection was an adaptation [19, 23] of the scanning in a fixed transect protocol [22], and as it was proposed, we delimited a 100-stretch at the stream margins, subdivide into 20 segments measuring 5 meters each (Fig 2). The adult specimens were collected using an entomological handnet (40 cm Ø, 65 cm deep, with an aluminum handle 90 cm long) during the one hour, always by the same collector, accompanied by an assistant (observer). The dragonflies were collected under the license SISBIO 14.457–1 issued by Brazilian environmental authorities (ICMBio) and deposited in the Alexander James Ratter Zoobotany collection, Mato Grosso State University, Nova Xavantina campus.

We delimited three transects (P1, P2, P3) for each stream, separated by 500 meters. To assess what is the best time to sample Odonata, we selected a sampling site in intermediate stretches of each stream (CRJC, CRB, and CRCU), each site was sampled (scanned) in three different hours: at 10:00 a.m.; 12 p.m., and 2 p.m. To evaluate how many days spent sampling the same site would represent Odonata species richness and composition well, we replicated treatment (I) twice more, performed at ten-day intervals. To determine what would be the number of segments (area size) needed to sample Odonata species richness and composition, we performed in all streams a 100 m sampling (P1 –the upper portion of the stream), a 200 m sample (P2 –an intermediate portion of the stream) and a 300 m sample (P3—downstream) (Fig 2).

After we collected the specimens, we performed all procedures proposed by Lencioni [34]. We identified all individuals using a stereomicroscope and specific taxonomic keys [9, 34, 35]. The specimens were deposited at the James Alexander Ratter zoo botanical collection, State University of Mato Grosso, Nova Xavantina campus.

### Data analysis

Species sampling is not always an easy task and each community has its peculiarities, whether due to the presence of rare species or with low detectability, causing possible sub-samples of the real diversity [36]. Due to this problem, in our study, we evaluated the efficiency of our methodology in capturing the existing biodiversity by analyzing the Sample coverage method [37]. In order to ensure that in each sampled transect it satisfactorily represents the diversity of species in each location. This method assesses sampling effectiveness at each sampling unit based on species abundance. We consider a limit of at least 60% of sample coverage. Therefore, all our transects were maintained in the analyzes (S1 Fig).

#### Richness

Due to the difficulties of sampling all the species existing in an area, we use the First-order Jackknife technique to estimate the number of species [38]. We estimate the richness for each of our treatments using the transects segments collected in the different streams as pseudo-replicates, thus totaling 180 pseudo-replicates per treatment. Additionally, we used a confidence interval (95%) associated with the standard deviation of the richness estimates by category for comparisons, that is, the richness of the treatments will only be different if the limits of the confidence intervals do not overlap the central value (richness) other categories. This technique allows us to test the variation in richness predicted.

We tested our predictions using three data treatments: Treatment (I)–to determinate when is the best time to sample odonates (i)–we compared richness estimates with the different sampling hours (10:00 a.m., 12 p.m., and 2 p.m.); Treatment (II)—evaluate how many collections over time are necessary to assess Odonata diversity in a representative way (ii)–we estimated the richness of species collected in the first sampling, then of species from 1+2 samplings, and finally from 1+2+3 samplings. After they were estimated, we compared the values; Treatment (III)–how many transects are necessary to sample a community–we compared the estimated richness from species collected in all P1 (= 100 m), P1+P2 (= 200 m), and P1+P2+P3 (300 m).

#### Composition

We assessed our predictions about the variation in composition by performing Principal Coordinate Analyses (PCoA). To test if there was a difference in species composition concerning the time of activity, sampling effort, and size of the sampling transect, we performed a Multivariate Permutation Analysis (PERMANOVA) [39], both analyzes using log-transformed species abundance (log+1) and Bray-Curtis dissimilarity [40]. We considered transects as samples, a randomization of 999 permutations with a significance level of p>0.05. All analyses were performed in the R environment, using the vegan package [41].

## Results

### Community structure

**Fig 3:**
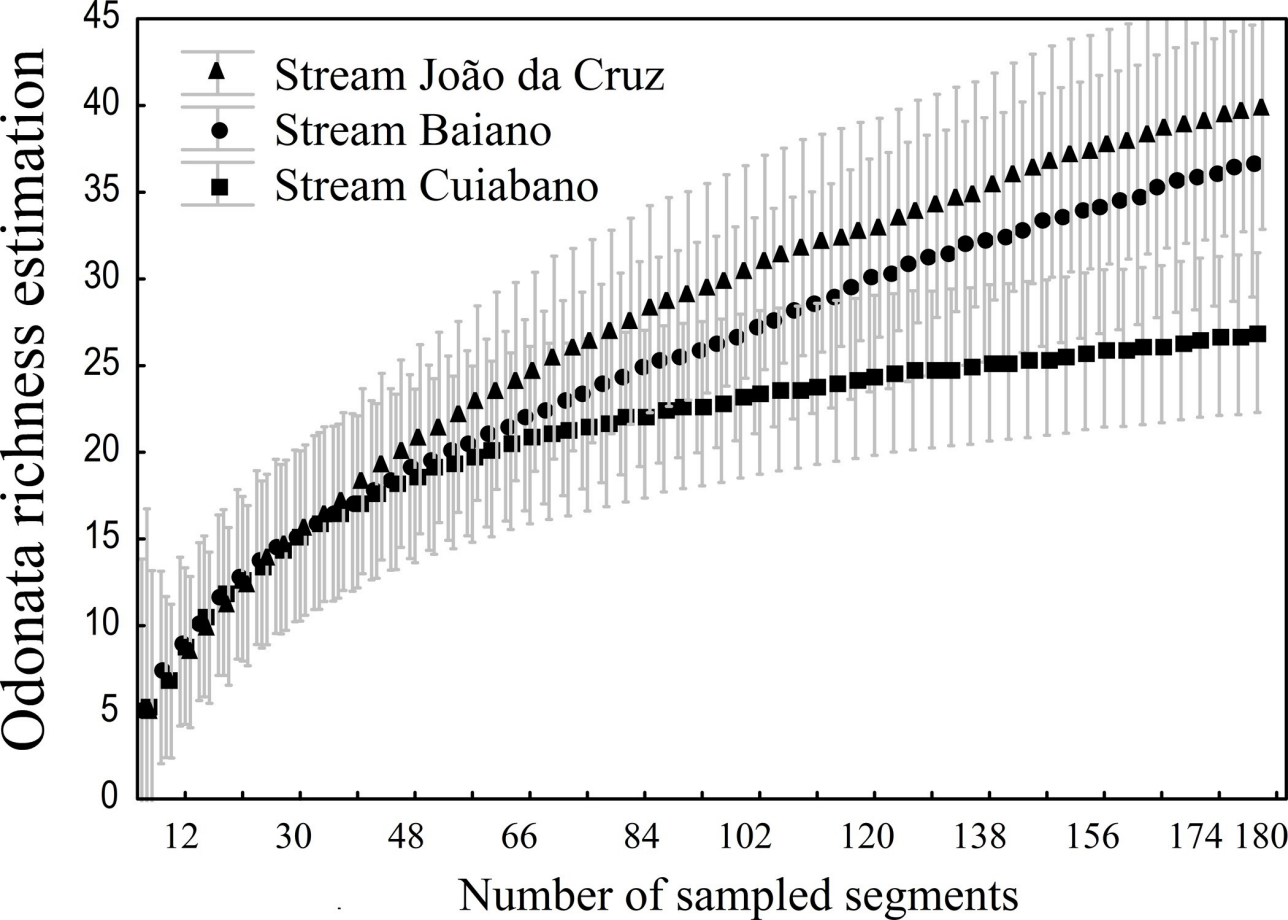
Estimated richness (jackknife) of Odonata. The sampling curve was based on the number of segments sampled in three streams at the Brazilian Cerrado (bars represent a 95% confidence interval). As noted, the curve has stabilized, thus showing a representative sampling of the dragonfly fauna in these locations.

We collected 661 Odonata adult individuals, from four families: Coenagrionidae (15 spp.), Libellulidae (22 spp.), Calopterygidae (4 spp.), and Aeshnidae (2 spp.); 26 genera and 43 species (see more in S1 Table). From the genera sampled, *Argia* had the highest number of individuals (n = 385). The stream with the highest species richness was João da Cruz, with 27 observed species (n = 248) and 40 (±6.92) estimated species (sampling efficiency = 68%), followed by the Baiano stream, with 25 observed species (n = 183) and 36.8 (±7.84) estimated species (68%), and, finally, the Cuiabano stream had 21 observed species (n = 230), and 26.9 (±4.60) estimated species (78%) (Fig 3).

### Does the time of collection influence the sampling of Odonata species richness and composition?

**Fig 4:**
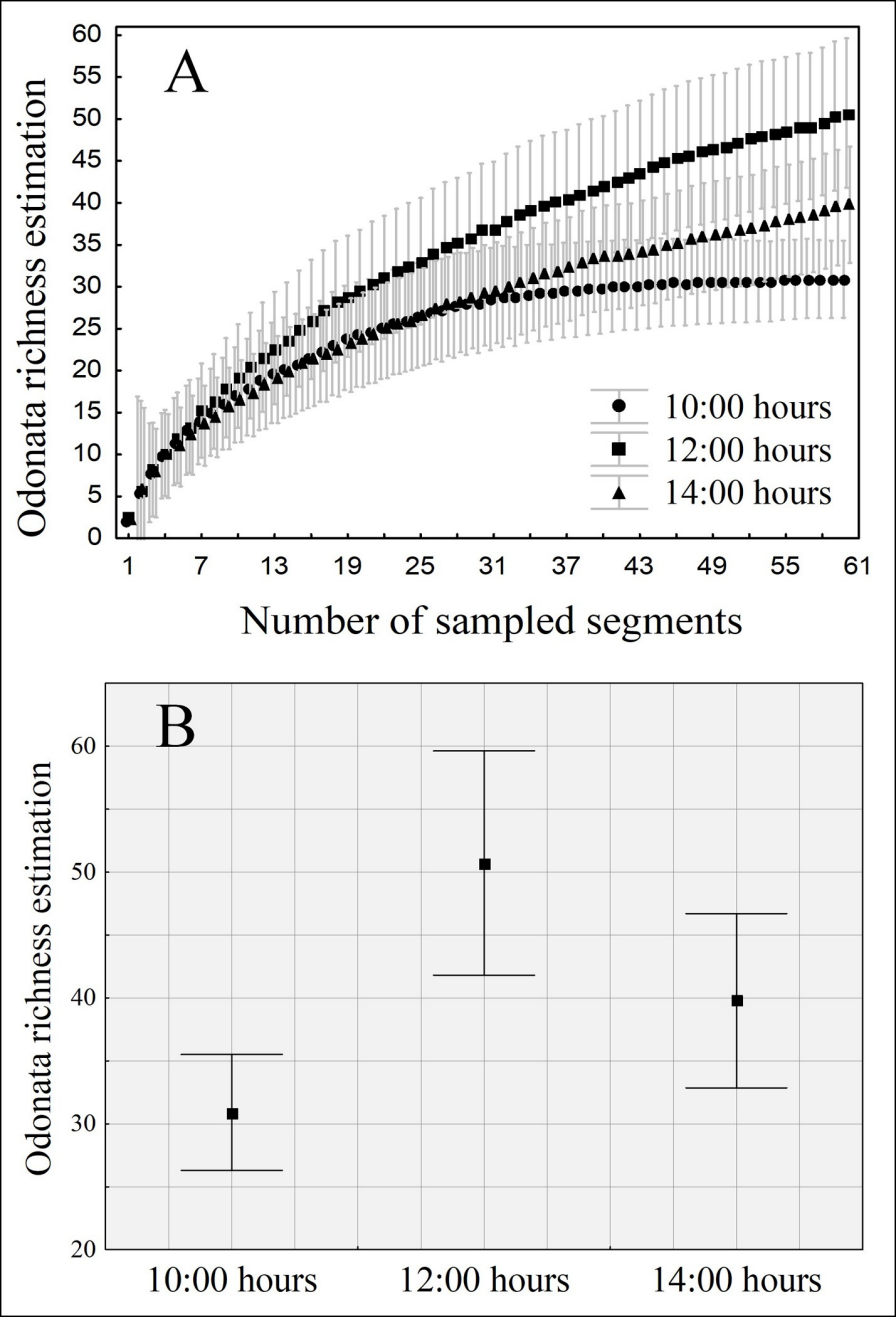
Estimated richness (jackknife) of Odonata. (A) the accumulation of richness over the sampled segments and (B) a 95% confidence interval around the estimated species average, both plotted according to the hours of collection.

As expected, the best time to sample the richness of odonates was during noon, once, at this hour, we estimated a mean sampling of 51 species (IC ± 8,9). The second-best time to sample was at 2:00 p.m., with an estimated richness of 40 species (IC ± 6,92). Finally, the hour with the lowest richness was at 10:00 a.m., with an estimated richness of 31 species (± 4,60) (Table 1) (Fig 4). Thus, samplings performed at 12:00 a.m. had an average of 20 species more than samplings performed at 10:00 a.m., and 11 more than collections performed at 2:00 p.m. Samplings performed at 2:00 p.m. had an average of 9 species more than the ones performed at 10:00. Thus, the highest species richness is obtained when samplings are performed at 12:00 a.m., then at 2:00 p.m., and the lowest richness is measured at 10:00 a.m. This difference is significant once the confidence interval of one group does not overlap the mean value of the other group (Fig 4).

**Table 1:** Estimated and observed richness (Odonata).

Treatment	Observed richness			Observed abundance		
	Mean	Deviation	Total	Mean	Deviation	Total
**(i) Sample time**						
10:00	13.6	3.5	25	105.3	45.9	316
12:00	15.7	4.5	34	84.3	11.8	253
14:00	14	1.7	27	74.3	37.9	223
**(ii) N° of samples**						
1 sample	11	1.7	21	77.7	21.6	233
2 sample	12.7	4	26	59.7	5.8	179
3 sample	14.3	5.1	28	56.3	11	169
**(iii) Transect**						
100 m	10	3.8	15	32	19.1	96
200 m	13	5.1	17	54.7	20.6	164
300 m	14.7	6	21	83.3	16.5	250

Mean values, standard deviation, and total observed species richness and abundance sampled on the different treatments (I, II, III).

Of the 43 species found in samples of treatment I, 15 were registered at all three sampling hours, 13 were recorded in two hours and 15 species were registered in one sampling hour. At 12:00 p.m. was the time that had the highest occurrence of exclusive species with eight records, followed by 2:00 p.m. with four species, and 10:00 a.m. with three species. When analyzing community composition, considering dragonflies collected at 10:00 a.m., we sampled 58.14% (25/43 spp.) of total diversity. Collections made at 2:00 p.m. represented 62.79% (27/43 spp.) and collections performed only at 12:00 p.m. represented 79.07% (34/43 spp.) of total diversity. However, there was no significant difference in composition between the collection hours (PseudoF = 0.540; p = 0.913, d.f. = 32), confirming what we expected in prediction (i) (S2 Fig).

### How many collections are necessary to sample Odonata species richness and composition?

**Fig 5:**
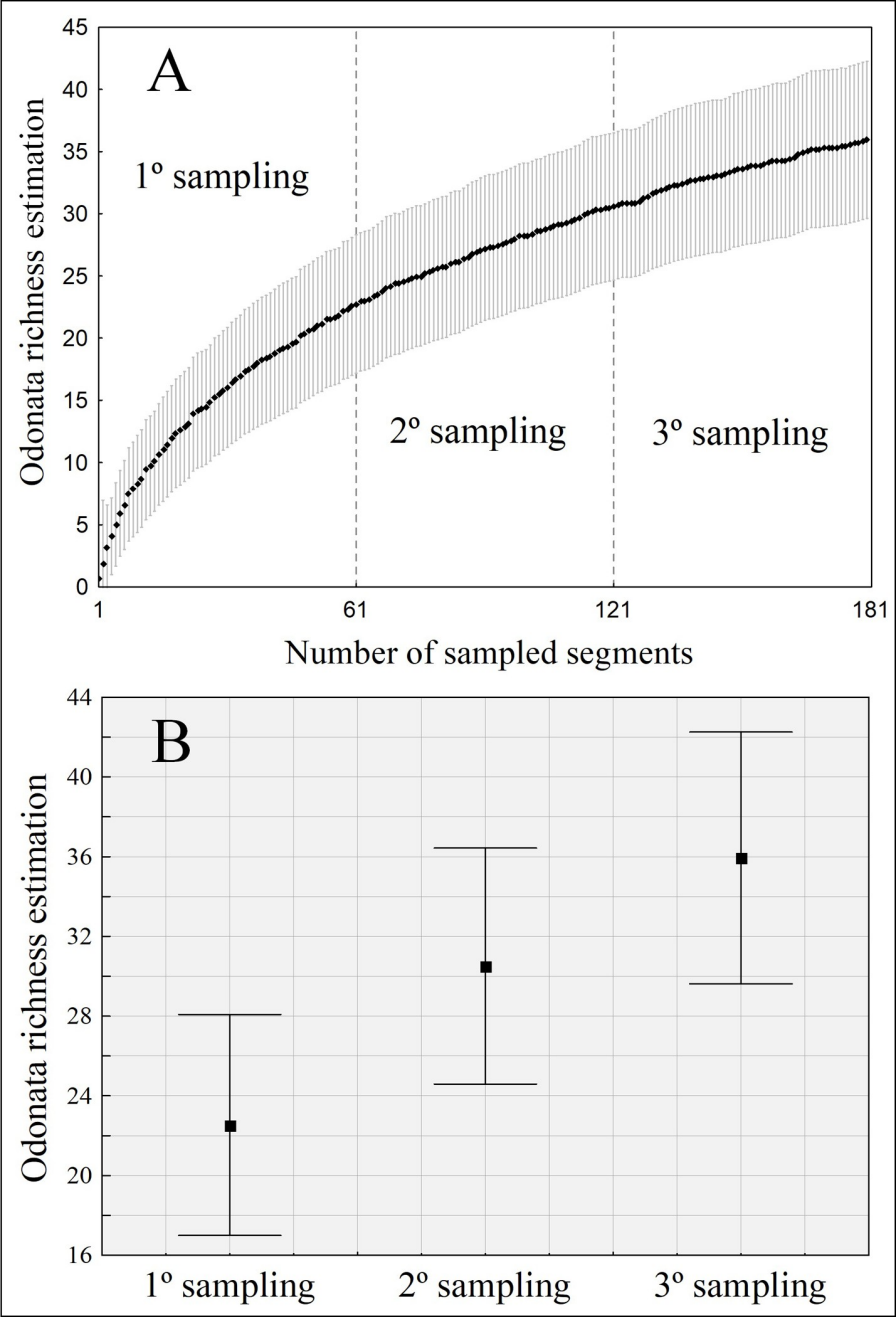
Estimated richness (jackknife) of Odonata. (A) the accumulation of richness over the sampled segments and (B) a 95% confidence interval around the estimated species average per treatment.

When sampling only once the species richness, we estimated an average of 22.5 species (±5.5). However, by sampling the same site a second time, we estimated an average of 30.5 species (±5.9), that is, an increase of seven species (Table 1). Therefore, when comparing Odonata estimated richness, we show there was a significant increase in the number of species between the first and second sampling, thus not corroborating prediction (ii) (Fig 5). When sampling the same site, a third time, we estimated an average of 36 species (±6.3), an average increase of 6 species. However, this increase was not significant. Thus, if we sampled each site only once, the species richness would represent only 51.22% (21 species) of total diversity. Additionally, in case the samplings were performed in two days, the fauna diversity would represent 75.61% (30.5) of local diversity, thus indicating that only two samplings are necessary to achieve a good representation of Odonata richness.

Regarding species composition, as expected by prediction (ii), there was no difference when the site was sampled more than once (Pseudo-F = 1.542; d.f. = 32; p = 0.107), showing only one sample is enough to represent Odonata species composition (S2 Fig).

### How many transects should be delimited to sample Odonata species richness and composition?

**Fig 6:**
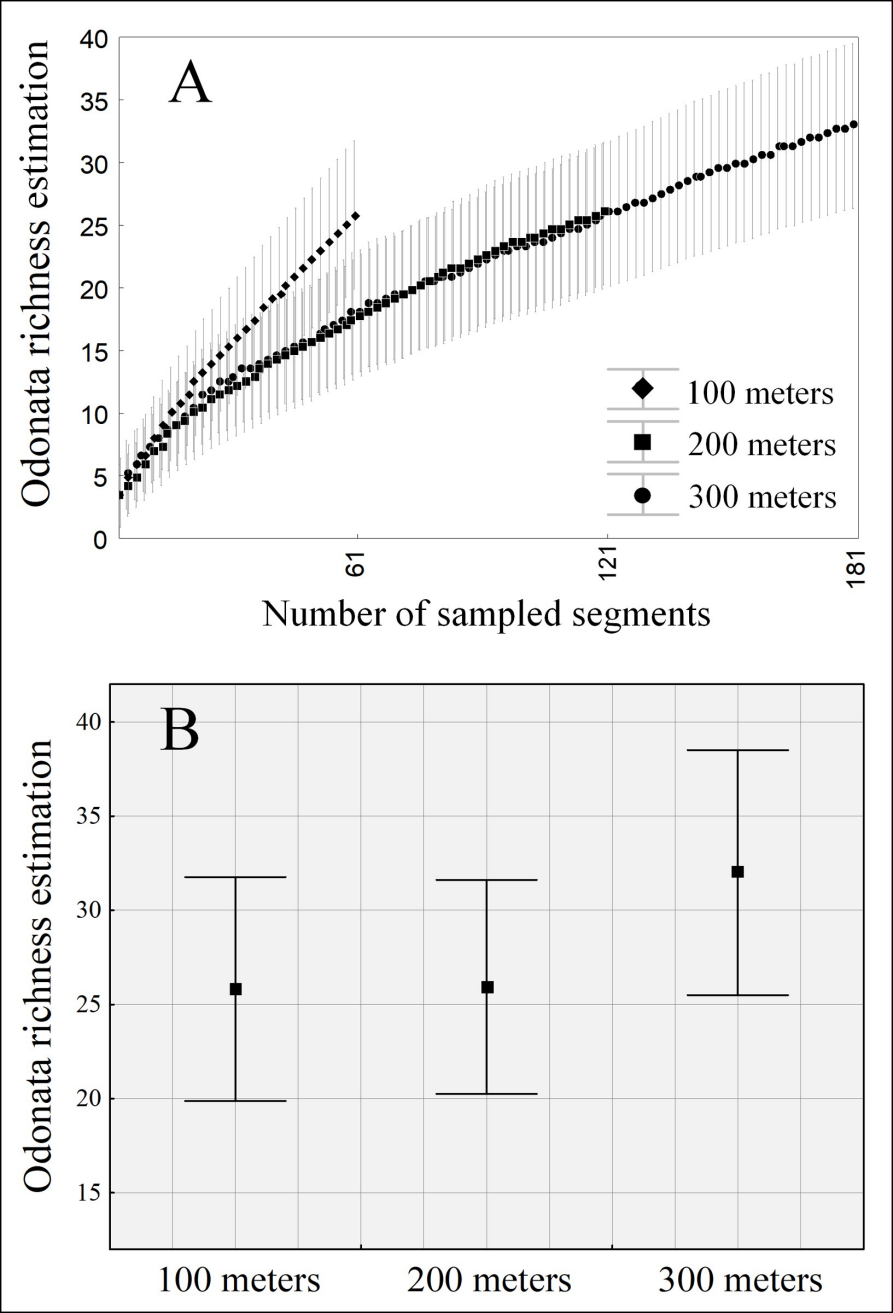
Estimated richness (jackknife) of Odonata. (A) All sites, considering the number of meters sampled and (B) mean estimated richness and the respective confidence interval (95%).

We did not evidence a gradual increase in species richness as the amount of sampled transects increases. When analyzing only the P1 of each stream, we estimate richness of approximately 26 species (±5.9). When analyzing the richness of P1+P2, we also estimated a richness of 26 species (±5.6). Finally, when P3 was added to the analyses, we estimated 32 species (±6.5) (Table 1), but those differences were not statistically significant (Fig 6).

Concerning species composition, of 21 species collected in treatment III, when considering the amount of transects sampled, only P1 (100 meters), we sampled 71,43% (15/21 spp.) of local diversity, while in P2 (200 meters), we sampled 80,95% (17/21 spp.) of local diversity. Finally, we reached 100% when considering all sampling sites (300 meters). When it comes to species composition, using 100, 200 or 300 meters of sampling did not show any significant difference (Pseudo f = 1.107; d.f. = 32; p = 0.391). Thus, it is enough to sample in 100 meters to capture the diversity of species inhabiting the streams (S2 Fig).

### When and how to collect Odonata adults?

**Fig 7:**
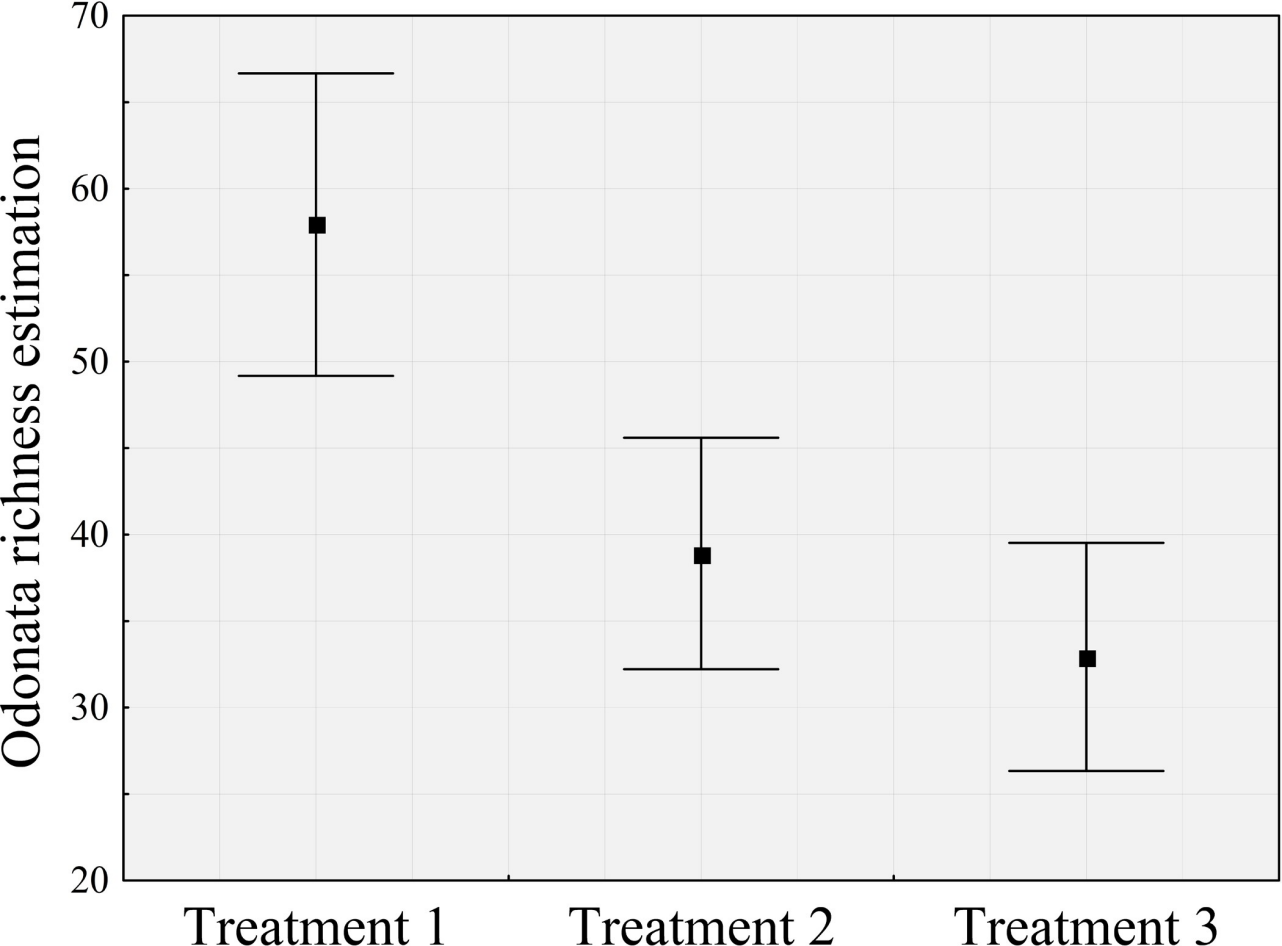
Comparison of the estimated richness (jackknife). The Odonata species were captured by treatment I (time), II (number of samples), and III (sample area).

When species richness was compared between treatments, using the method of confidence interval inference, the treatment I (hour) showed an average of 16.2 species more than treatment III (sample area) and 22 more than treatment II (number of samplings) (Fig 7). Thus, these result shows that a single sample in 100 m of transect, preferably at noon, can capture the existing diversity well. Thus, these results show that a single sample in 100 m of transect, preferably at noon, can capture the existing diversity well.

## Discussion

Assessing biodiversity is one of the greatest challenges for scientists, especially in regions of high variation, such as the Tropical region [36]. The methodology applied in the present study proved to be efficient, and the richness assessed was similar to what was found in studies at the regional and local scale [6, 17, 30, 42, 43]. We observed variation in species richness patterns, especially regarding sampling time, evidencing noon was the best time to sample dragonflies. This pattern is directly linked to the physiological restrictions of the group and the variation in temperature throughout the day. The majority of tropical odonates are ectotherms, warming up through constant heat exchange with the surrounding environment [5]. Additionally, temperature varies throughout the day due to the different levels of light entry in the stream, where gradual warming of the stream towards a peak is expected, which generally occurs at noon, decreasing after that time. Thus, as observed, due to the increased availability of solar energy and the ecophysiological requirements of the group, we expected dragonflies would be more active and next to water bodies in the hours following noon, which allowed for more efficient sampling. The contrary also applies, as at 10:00 a.m. the sunlight has not yet reached the stream due to the dense riparian forest. Some dragonflies have not started their activities yet, remaining settled in a lethargic state, making it difficult for them to be seen by the collector; or are in search of abundant solar radiation, becoming dispersed atop tree canopies [44], which is a location out of reach for the collector.

We also show the obtained species richness depends on the time of collection and the sampling effort. The higher the sampling effort, the higher the number of individuals collected, increasing the probability of collecting rare species, making the sampling more representative [45, 46]. More specifically, we show that only two samplings are enough to sample the local diversity. These results agree with sampling efforts applied in several ecological studies with the order Odonata [6, 19, 42, 47, 48].

Additionally, the ecological conditions on the day of the collection are not always favorable. For example, dragonflies act in the food web both as secondary consumers and as food resources. The abundance of species, and, therefore, their detectability, depends on food supply and predator rate in a given time window [2]. To further this ecological point of view, dragonflies are hosts of several parasite species [49] or suffer exclusion pressure for competing with invasive species [50], relationships that act directly in population density [44]. Furthermore, environmental conditions also change as time passes, and odonates are sensitive to this variation. For example, changes in community structure were observed due to anthropogenic change, mainly related to vegetation [29, 51, 52] and temperature [6, 11]. Given the above, it is possible that at the time of sampling, the absence of these influences is noticed, but they may be subtle or have occurred a few days before sampling, and the community might still be under the influence or recovering from them. Thus, once the possibility of spurious (whether seasonal, spatial, or ecological) phenomena is raised, it becomes even more evident the importance of (when possible) carrying out more than one sampling of Odonata in the same site.

Richness was not associated with the different spatial efforts employed, with no increase in richness as the area of sampled transects increased. Dragonflies are flying organisms with a high dispersal ability [10]. Perhaps the difference between 100, 200 and 300 meters is not enough to capture the change in species richness due to the easy dispersion of these insects along the stream, making it expensive to sample spaces larger than 100 meters. Moreover, Brasil et al. [53] showed that the alpha diversity of stream Odonata is practically constant, whereas the biggest change is in beta diversity among streams.

Dragonflies are distributed irregularly on the stream [54], being more frequent in areas with resources, for example, places with spots of light (formed by holes in the dense riparian forest) [51], areas with perches [55] or specific substrates for oviposition [31]. However, despite this kind of distribution and the natural environmental variation found in aquatic systems, the 100 m stretch assessment at the streams was efficient, as we did not find significant differences in species composition in bigger transects. Furthermore, the flying ability may also explain why 1, 2, or 3 transects (500 m apart from each other) could not capture potential changes in composition. Therefore, perhaps the spatial variation captured by the extent of the transects analyzed in this study was not enough to contemplate species with specific environmental requirements. The same reasoning can be considered to explain the similarity in composition found on the different sampling days, however, in this case, from a temporal perspective. Within the order Odonata, it is common to observe adult life cycles that exceed weeks, and may, in larger species, reach up to 4 months [44]. Once we sampled sites every 10 days, totaling one month, there is a possibility that we do not have a sufficient period to observe a potential replacement of species.

Recently, Buss et al. [56] assessed 13 sampling protocols for macroinvertebrates used in different continents. Most protocols that use a fixed number of samples distribute them according to the variation in the amount of substrate, that is, high heterogeneity requires increased sampling. However, for dragonflies, we show that 100 meters of the stream to sample is enough to capture the environmental heterogeneity of the sites and, with that, contemplate species with different environmental requirements. Traditionally, most ecological studies standardize sampling effort by the number of sampling units. Some studies stipulate that in quick stream assessments, the standardization of effort using the number of individuals is efficient, suggesting a fixed number of 100 individuals [57]. A more reasonable assessment is suggested to sample from 200 to 300 macroinvertebrate individuals [58]. However, we believe this type of standardization per individual might not be very efficient for Odonata since the observed abundance in each water body is not high enough. Another problem is that Odonata often has a clustered distribution [55], which would probably affect standardization through abundance.

Finally, we did not find any difference in composition between the evaluated hours. In addition to those dragonflies physiologically categorized as ectotherms, there is also the group of endotherms, that depend less on the temperature of the environment [5]. Generally, this group performs daily activities at hours that are different from those sampled by us, being more active during the twilight period [20, 44]. In this case, in the recommended and traditionally established sampling between the interval of 10:00 to 15:00 hours [19, 23], only a specific group of dragonflies, mainly ectotherms, will be at full peak activity and, therefore, susceptible to capture. Supporting this hypothesis, Almeida et al. [20] showed a strong tendency to collect different species by using other methods (e.g. malaise and light trap) that cover the twilight and night period. Thus, if the intention is to inventory as many species as possible, including those belonging to that specific physiological group, a methodological flaw in the timetable traditionally adopted when using this protocol is evident.

## Conclusions

Measuring species richness has never been an easy task. For biological inventories to become useful in making conservation decisions or advancing science, it is essential to apply a well-developed, well-tested, and of course, efficient sampling protocol. Here, we assessed the scanning in fixed areas method, widely used in Brazilian studies with Odonata. We show the best efficiency is reached with samples performed at the interval between 12 and 2 p.m. Additionally, we show that only 100 meters of the transect is enough to represent the community well. Thus, we show a sampling efficiency concerning time and spatial effort traditionally adopted by many ecological studies using Odonata. We also found that only two samples, over a small-time window, are sufficient to obtain a good representation of the fauna. Finally, by comparing species composition between the different treatments, we show that the time and effort of collection, whether temporal or spatial, have no direct implications for capturing the local species pool. However, we highlight and discuss some possible methodological flaws when using this protocol to inventory specific Odonata groups. We found that the protocol is efficient if the conditions of time, climate and space are considered. We believe the results obtained are fundamental in conducting future studies with the group and aid of conservationist measures, such as environmental monitoring using the order Odonata.

## Supporting information

S1 Table Occurrence and abundance of adult Odonata species in the Ribeirão Antártico Basin, MT, 2008. (CRJC- João da Cruz Stream; CRB- Baiano Stream; CRCU- Cuiabano Stream).
(DOCX)

S1 Fig Sample coverage. We consider a limit of at least 60% of sample coverage. Therefore, all our transects were maintained in the analyzes.
(DOCX)

S2 Fig Variation in Odonata species composition. We order considering time (T1), temporal (T2) and spatial (T3) sampling efforts.
(DOCX)
