# Supplementary material for: Sampling efficiency of a protocol to measure Odonata diversity in tropical streams
Source: PLoS One. 2021 Mar 9;16(3):e0248216. doi: 10.1371/journal.pone.0248216 (PMC7942985; doi:10.1371/journal.pone.0248216)
Supplement: S1 Table — (CRJC- João da Cruz Stream; CRB- Baiano Stream; CRCU- Cuiabano Stream). (DOCX) [file pone.0248216.s001.docx]

**S1 Table. Occurrence and abundance of adult Odonata species in the Ribeirão Antártico Basin, MT, 2008.** (CRJC- João da Cruz Stream; CRB- Baiano Stream; CRCU- Cuiabano Stream).

| **SPECIES** | **CRJC** | **CRB** | **CRCU** | **TOTAL** |
| --- | --- | --- | --- | --- |
| **Zygoptera** |  |  |  |  |
| *1-Acanthagrion ascendens* Calvert, 1909 | 0 | 0 | 6 | 6 |
| *2-Acanthagrion jessei* Leonard, 1977 | 0 | 0 | 2 | 2 |
| *3-Argia reclusa* Selys, 1865 | 2 | 6 | 80 | 88 |
| *4-Argia eliptica* Selys, 1865 | 1 | 0 | 0 | 1 |
| *5-Argia insipida* Hagen *in* Selys, 1865 | 1 | 0 | 0 | 1 |
| *6-Argia mollis* Hagen *in* Selys, 1865 | 4 | 6 | 0 | 10 |
| *7-Argia tinctipennis* Selys, 1865 | 146 | 59 | 80 | 285 |
| *8-Epipleoneura metallica* Rácenis, 1955 | 8 | 1 | 0 | 9 |
| *9-Epipleoneura williamsoni* Santos, 1957 | 2 | 1 | 0 | 3 |
| *10-Hetaerina laesa* Hagen *in* Selys, 1853 | 5 | 17 | 2 | 24 |
| *11-Hetaerina rosea* Selys, 1853 | 4 | 3 | 11 | 18 |
| *12-Metaleptobasis selysi* Santos, 1956 | 1 | 0 | 2 | 3 |
| *13-Mnesarete fuscibasis* (Calvert, 1909) | 1 | 0 | 0 | 1 |
| *14-Mnesarete guttifera* (Selys, 1873) | 10 | 5 | 1 | 16 |
| *15-Oxyagrion chapadense* Costa, 1978 | 1 | 0 | 2 | 3 |
| *16-Phoenicagrion karaja* Machado, 2010 | 1 | 2 | 0 | 3 |
| *17-Protoneura tenuis* Selys, 1860 | 0 | 0 | 2 | 2 |
| *18-Telebasis racenisi* Bick & Bick, 1995 | 1 | 1 | 0 | 2 |
| *19-Telebasis gigantea* Daigle 2002 | 30 | 53 | 11 | 94 |
| **Anisoptera** |  |  |  |  |
| *20-Aeshna williamsoniana* Calvert, 1905 | 0 | 1 | 0 | 1 |
| *21-Anatya normalis* Calvert, 1899 | 2 | 8 | 0 | 10 |
| *22-Brechmorhoga travassosi* Santos, 1946 | 0 | 1 | 0 | 1 |
| *23-Diastatops pullata* (Burmeister, 1839) | 1 | 2 | 0 | 3 |
| *24-Dythemis multipunctata* Kirby, 1894 | 0 | 0 | 4 | 4 |
| *25-Elga leptostyla* Ris, 1909 | 2 | 0 | 8 | 10 |
| *26-Erythemis haematogastra* (Burmeister, 1839) | 0 | 1 | 0 | 1 |
| *27-Gynacantha chelifera* McLachlan, 1895 | 0 | 1 | 0 | 1 |
| *28-Macrothemis flavescens* (Kirby, 1897) | 1 | 1 | 0 | 2 |
| *29-Macrothemis heteronycha* (Calvert, 1909) | 0 | 0 | 2 | 2 |
| *30-Micrathyria coropinae* Geijskes, 1963 | 0 | 0 | 10 | 10 |
| *31-Micrathyria hippolyte* Ris, 1911 | 2 | 8 | 1 | 11 |
| *32-Micrathyria mengeri* Ris, 1919 | 1 | 0 | 0 | 1 |
| *33-Micrathyria occipita* Westfall, 1992 | 1 | 0 | 0 | 1 |
| *34-Micrathyria pseudhypodidyma* Costa *et al*., 2002 | 1 | 0 | 1 | 2 |
| *35-Micrathyria spinifera* Calvert, 1909 | 0 | 1 | 0 | 1 |
| *36-Nephepeltia leonardina* Rácenis, 1953 | 0 | 0 | 1 | 1 |
| *37-Oligocla abbreviata abbreviata* (Rambur, 1842) | 0 | 0 | 1 | 1 |
| *38-Orthemis ambirufa* Calvert, 1909 | 2 | 3 | 0 | 5 |
| *39-Orthemis anthracina* De Marmels, 1989 | 1 | 2 | 0 | 3 |
| *40-Staurophlebia reticulata* (Burmeister, 1839) | 0 | 1 | 0 | 1 |
| *41-Uracis siemensi* Kirby, 1897 | 0 | 1 | 0 | 1 |
| *42-Zenithoptera fasciata* (Linnaeus, 1758) | 14 | 1 | 1 | 16 |
| *43-Zenithoptera viola* Ris, 1910 | 0 | 0 | 1 | 1 |
| **Total individuals** | **246** | **186** | **229** | **661** |
| **Total species** | **27** | **25** | **21** | **43** |
