# Supplementary material for: Sampling efficiency of a protocol to measure Odonata diversity in tropical streams
Source: PLoS One. 2021 Mar 9;16(3):e0248216. doi: 10.1371/journal.pone.0248216 (PMC7942985; doi:10.1371/journal.pone.0248216)
Supplement: S1 Fig — We consider a limit of at least 60% of sample coverage. Therefore, all our transects were maintained in the analyzes. (DOCX) [file pone.0248216.s002.docx]

**
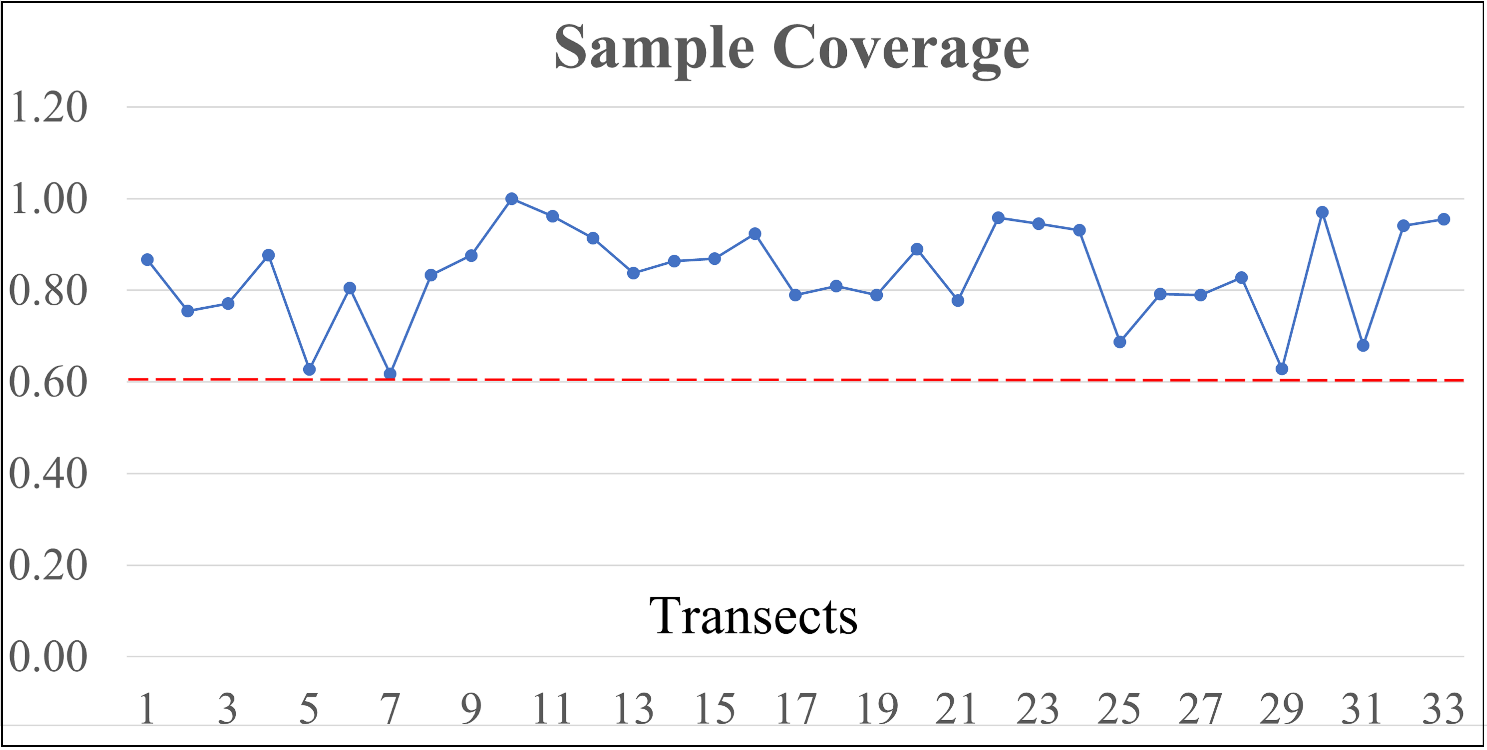
**

**S1 Figure. Sample coverage.** We consider a limit of at least 60% of sample coverage. Therefore, all our transects were maintained in the analyzes
