# Supplementary material for: Sampling efficiency of a protocol to measure Odonata diversity in tropical streams
Source: PLoS One. 2021 Mar 9;16(3):e0248216. doi: 10.1371/journal.pone.0248216 (PMC7942985; doi:10.1371/journal.pone.0248216)
Supplement: S2 Fig — We order considering time (T1), temporal (T2) and spatial (T3) sampling efforts. (DOCX) [file pone.0248216.s003.docx]

**
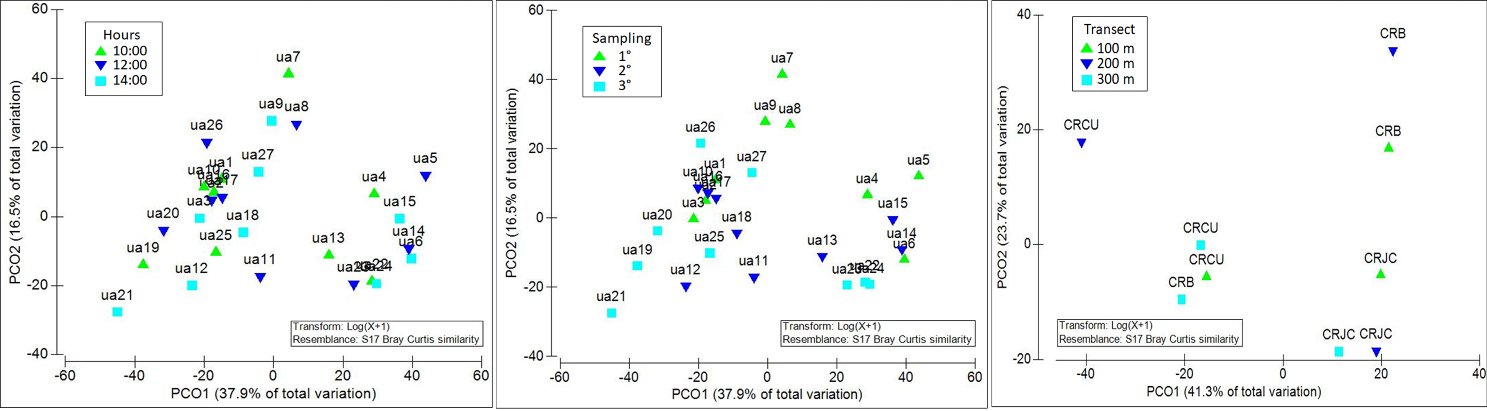
S2 Figure. Variation in Odonata species composition.** We order considering time (T1), temporal (T2) and spatial (T3) sampling efforts.
